# Integrating reinforcement learning and serious games to support people with rare genetic diseases and neurodevelopmental disorders: outcomes on parents and caregivers

**DOI:** 10.3389/fpsyg.2024.1372769

**Published:** 2024-04-05

**Authors:** Fabrizio Stasolla, Khalida Akbar, Anna Passaro, Mirella Dragone, Mariacarla Di Gioia, Antonio Zullo

**Affiliations:** ^1^University “Giustino Fortunato”, Benevento, Italy; ^2^Faculty of Health Sciences, Durban University of Technology, Durban, South Africa; ^3^MANCOSA, Research Doctorate, Durban, South Africa; ^4^Universitas Mercatorum, Rome, Italy

**Keywords:** adaptive responses, artificial intelligence, cognitive abilities, neurodevelopmental disorders, reinforcement learning, serious games, rare genetic diseases

## Introduction

Individuals grappling with rare genetic diseases, such as Angelman, Cornelia de Lange, Fragile X, and Rett syndromes, confront formidable challenges in navigating their daily environments. In addition to intellectual disabilities, communication deficits, and sensorial impairments, these individuals often contend with profound motor disorders. This complex situation not only seriously compromises their quality of life but also places an increased burden on both caregivers and families (Krath et al., 2021).

To tackle these challenges, technological interventions have emerged as promising solutions. Serious games and utilizing new technologies, offer immersive and entertaining experiences with educational, diagnostic, and rehabilitative purposes. Increasingly, artificial intelligence-based programs, particularly those employing reinforcement learning, have been adopted. This sophisticated approach involves an artificial intelligent agent continuously interacting with a participant's performance, adapting the complexity or difficulty of tasks or activities in real-time (Krath et al., 2021; Liu et al., 2022). This personalized adaptation ensures optimal user engagement and effectiveness. In this paper, we advocate for the integration of serious games and reinforcement learning to serve both assessment and rehabilitative goals. This combined approach may present a tailored solution to promote adaptive responding in individuals with rare genetic diseases. We explore various domains, including cognitive skills with executive functions, communication abilities, and managing challenging behaviors. We acknowledge the profound effects on participants' quality of life, providing illustrative examples to underscore our points. Our innovative approach combines gamification with Piaget's theory of cognitive development, categorizing it within a cognitive framework that fosters new adaptive skills (Robb et al., 2019).

Recently, Stasolla and Di Gioia (2023), and Stasolla et al. (2023a,b) argued on the combined use of virtual reality, extended reality, and reinforcement learning for assessment and rehabilitative objectives. The above mentioned contributions detailed cognitive rehabilitation for mild neurocognitive impairments, and healthcare and reduction of social anxiety in fragile X population, respectively. We critically discuss the implications of this integrated approach and offer insights for future research perspectives. Lastly, we delve into a critical analysis of the combined technologies in clinical settings, emphasizing the pressing need for technological interventions. This paper underscores the potential of serious games and reinforcement learning as innovative solutions to address the multifaceted challenges faced by individuals with rare genetic diseases, laying the groundwork for their integration into assessment and rehabilitation strategies.

## Revolutionizing intervention strategies through the fusion of serious games and AI for rare genetic diseases and neurodevelopmental disorders

Within the complex domain of rare genetic diseases and neurodevelopmental disorders, patients contend with a multitude of obstacles that transcend the mere physical manifestations of their ailments. The intricate interweaving of intellectual, communicative, and motor abilities within these disorders has a profound effect on the individuals afflicted and places an added strain on their careers. This segment explores the critical necessity for technological interventions, with a particular emphasis on the inventive collaboration between serious games and artificial intelligence (AI), in order to tackle the distinct requirements of individuals confronted with these intricate challenges. Angelman, Cornelia de Lange, Fragile X, and Rett syndromes are examples of rare genetic disorders that comprise a spectrum of conditions, each of which presents affected individuals with unique challenges (Sommese and Corrado, 2021). Neurodevelopmental disorders exacerbate these difficulties, resulting in a multifaceted fabric that requires interventions that are both dynamic and adaptable. Within this particular framework, technology arises as a symbol of optimism, providing opportunities for intervention that surpass conventional approaches and accommodate the varied requirements of individuals navigating this complex terrain. Contemporary developmental cognitive science has progressed beyond Piaget's insights, identifying constructivism with the conceptual changes best described by the theory-theory of development exemplify these changes in intuitive biology, showcasing a shift from an initial theory where children identify life with animals as causal and intentional agents to a later vitalist theory (Bascandziev et al., 2018). This shift is seen as evidence for the constructivist hypothesis, strengthened by the absence of evidence supporting innate concepts within vitalist biology. These insights underscore the need for interventions that address the multifaceted characteristics of these disorders. The severe consequences that rare genetic diseases and neurodevelopmental disorders impose on a wide range of human functions emphasize the urgent requirement for technological interventions (Zylka, 2020). Conventional methodologies frequently prove inadequate in offering flexible and intricate resolutions demanded by the multifaceted characteristics of these disorders. Technological interventions serve as a means of bridging this divide, providing not only assistance but also a paradigm shift that corresponds with the distinctive obstacles presented by these circumstances (Fitzgerald and Gallagher, 2022). Serious games, being a technological advancement, distinguish themselves as a versatile instrument with numerous objectives. These encompass education, rehabilitation, and diagnostics, rendering them indispensable in tackling the multifaceted array of obstacles linked to rare genetic diseases and neurodevelopmental disorders. In contrast to traditional approaches, serious games integrate education, engagement, and immersion into a gamified structure, thereby offering people a pleasurable and interactive channel through which to gain knowledge and grow. A paradigm shift in the approach to education and intervention is exemplified by serious games (Vacca et al., 2023).

When considering rare genetic diseases and neurodevelopmental disorders, these games provide more than just amusement. They function as immersive platforms that are specifically engineered to tackle the unique challenges and requirements associated with these conditions. Serious games possess significant potential as educational instruments due to their ability to captivate individuals in a gamified learning environment, surpassing the limitations of conventional pedagogical methods. By integrating educational materials into the framework of a game, the process of learning is intrinsically enlivened and becomes more interactive, thereby cultivating a favorable learning environment for individuals who are facing cognitive difficulties. Within the domain of diagnostics, serious games offer an innovative method for evaluating motor, communicative, and cognitive abilities. The real-time observation and data collection capabilities of these games, which are interactive in nature, provide clinicians with invaluable insights into the strengths and weaknesses of each individual. The implementation of this gamified diagnostics approach not only optimizes the evaluation procedure but also offers a more captivating and easily navigable medium for individuals grappling with these disorders. Serious games have also brought about a revolution in rehabilitation, which is an essential component in the voyage of individuals with uncommon genetic diseases and neurodevelopmental disorders. By providing a gamified environment for therapeutic exercises, these games increase the engagement and motivation of rehabilitation. Active engagement is facilitated by the immersive qualities of serious games, which enhances the efficacy of rehabilitation interventions (Bukovšek et al., 2020; Jiang et al., 2022; Cervantes et al., 2023). Concurrently with the development of serious games, the incorporation of reinforcement learning, a form of artificial intelligence (AI), enhances the capabilities of technological interventions in this field. Artificial intelligence (AI) enhances the customization and flexibility of interventions, which is critical when it comes to attending to the varied requirements of people with uncommon genetic diseases and neurodevelopmental disorders. As an application of artificial intelligence, reinforcement learning infuses interventions with dynamism through the continuous modification of tasks and activities in response to individual progress. The ability to adapt in real-time guarantees that the degree of challenge corresponds to the specific capabilities of every participant, thereby providing a customized and flexible learning trajectory (Kleberg et al., 2023).

When considering rare genetic diseases and neurodevelopmental disorders, which are characterized by a broad range of abilities, reinforcement learning proves to be an indispensable element in customizing interventions for each affected individual. By combining reinforcement learning and serious activities, technology-assisted interventions reveal their full potential. The framework that results from the synergistic integration of these technologies surpasses conventional approaches in its flexibility and dynamism. By integrating reinforcement learning's flexibility and the gamified approaches to education, diagnostics, and rehabilitation found in serious games, a comprehensive and individualized intervention can be developed for those afflicted with neurodevelopmental disorders and rare genetic diseases. This integrated methodology transforms serious games into dynamic platforms for real-time assessment and rehabilitation, in addition to immersive learning tools. The implementation of reinforcement learning guarantees that interventions are modified to align with the advancements made by each individual, thereby cultivating a personalized and efficacious support system. Although the incorporation of reinforcement learning and serious games signifies a notable advancement in technology-supported interventions, the process remains ongoing. Further investigation is warranted to concentrate on enhancing and broadening the scope of this integrated methodology (Lopes and Lopes, 2022; Rodrigo-Yanguas et al., 2022). In order to ascertain the long-term effects of these interventions on cognitive, communicative, and motor abilities, longitudinal research is crucial. Furthermore, a comprehensive examination of the potential ethical ramifications associated with the integration of AI into interventions is imperative. Privacy considerations, consent procedures, and the wider societal ramifications of these technologies ought to be thoroughly examined in order to guarantee their ethical and responsible integration. Comparative research is warranted due to the adaptability of serious games and reinforcement learning in addressing a wide range of rare genetic diseases and neurodevelopmental disorders. Gaining insight into the efficacy of this integrative methodology across various conditions will aid in the enhancement and customization of interventions to correspond with the unique attributes of each disorder. In summary, technology-supported interventions, specifically those that incorporate reinforcement learning and serious games, symbolize a paradigm shift in how complex obstacles associated with uncommon genetic diseases and neurodevelopmental disorders are approached (Srinivasan and Shah, 2019). The convergence of reinforcement learning's adaptability and the gamified approaches to education, diagnostics, and rehabilitation found in serious games results in a framework for personalized and dynamic interventions. This groundbreaking collaboration not only presents an unprecedented strategy for education, diagnosis, and rehabilitation, but also has the capacity to fundamentally transform the assistance network for people afflicted with uncommon genetic disorders and neurodevelopmental conditions. An innovative advancement occurs with the combination of reinforcement learning and serious games; technology becomes a collaborator in delivering customized and efficacious interventions that promote individual empowerment and adaptation, as well as that of their support systems (Lussier-Desrochers et al., 2023).

### Comprehensive integration in addressing rare genetic diseases and neurodevelopmental disorders technologically

Serious games have emerged as a promising avenue for addressing the multifaceted challenges posed by rare genetic diseases and neurodevelopmental disorders. Leveraging computer technology with predictable and affect-free interfaces, serious games offer an engaging and enjoyable way to intervene in various aspects, including emotional recognition, social skills, and daily living skills. Currently, various approaches are utilized in the implementation of a Serious Game as Digital Therapeutics (DTs). These approaches may vary based on factors such as the patient category, the intended purpose, and potential requirements, including the need for data acquisition to monitor the patient's progress or provide feedback regarding game adaptation. Additionally, the underlying technologies employed in Serious Games as DTs play a crucial role in achieving the intended outcomes by enabling patients to interact with the elements of the game mechanics (Vacca et al., 2023). The development of game-based training platforms for behavioral interventions has gained momentum, particularly for individuals with autism. In the past decade, a plethora of serious games has been developed, catering to personalized training and cognitive assessments. These games, ranging from role-play to single-player modes, serve as powerful tools for enhancing emotional recognition and social skills. The integration of gamification strategies, coupled with Piaget's theory of cognitive development, facilitates the creation of a cognitive framework and the cultivation of novel adaptive abilities. This approach aligns with the aim of providing exceptionally tailored remedies to individuals with uncommon genetic disorders, promoting adaptive responses (Dong et al., 2022; Liang et al., 2022).

### Reinforcement learning and adaptive interventions

Alongside serious games, artificial intelligence, particularly reinforcement learning, has gained prominence in adapting interventions based on user performance. This section explores the potential of reinforcement learning as a component of artificial intelligence to enhance the adaptability of interventions for individuals facing rare genetic diseases and neurodevelopmental disorders. Reinforcement learning involves an artificial intelligent agent constantly interacting with the participant's performance, adapting the complexity or difficulty of tasks based on progress. This continuous adaptation ensures highly customized and tailored solutions, promoting adaptive responding in individuals with neurodevelopmental disorders. The integration of reinforcement learning in serious games represents a dynamic approach to rehabilitation and assessment, offering real-time adjustments to optimize the user experience (Zhou et al., 2020; Salgado-Cacho et al., 2022; Zhang et al., 2023).

### Clinical implications and future perspectives

This section critically discusses the potential clinical implications of integrating serious games and reinforcement learning in the context of rare genetic diseases and neurodevelopmental disorders. The exploration of various domains, including cognitive abilities, executive functions, communication skills, and challenging behavior management, highlights the comprehensive nature of this integrated methodology. Illustrative examples, informed by Piaget's theory of cognitive development, showcase the effectiveness of combining serious games and reinforcement learning for cognitive framework formation and adaptive skill cultivation. The critical analysis of the ramifications of this integration sheds light on significant avenues for future research. The proposed integration, as outlined in this opinion article, seeks to pave the way for individualized options that promote adaptive responding and improve the quality of life for individuals with rare genetic diseases. Therefore, the integration of serious games and reinforcement learning holds promising potential for addressing the challenges posed by rare genetic diseases and neurodevelopmental disorders. This combined approach offers a holistic and individualized solution, with implications for education, diagnostics, and rehabilitation. As technology continues to advance, further research in this domain is crucial to unlock the full potential of these integrated technologies in clinical settings (Huang et al., 2017).

## Integration of technologies for individualized solutions

This section advocates for the integration of serious games and reinforcement learning, emphasizing the individualized and adaptive nature of this combined approach. It explores its potential in targeting cognitive skills, communication abilities, and managing challenging behaviors, while considering its impact on participants quality of life. Within the domain of neurodevelopmental disorders and rare genetic diseases, patients frequently confront an array of obstacles that transcend the mere physical constraints inherent in their ailments. Intellectual disabilities, communication impairments, sensory deficits, and extensive motor disorders significantly diminish the quality of life of affected individuals. The burden of these challenges is borne by family members and caregivers, which requires the development of novel strategies to improve the quality of life for those impacted. The present paper argues in favor of a collaborative amalgamation of reinforcement learning and serious games in order to offer personalized and adaptable assistance to people dealing with neurodevelopmental disorders and uncommon genetic diseases. By combining various approaches, this method attempts to cater to the distinct requirements of every individual by providing a dynamic and customized intervention that surpasses conventional techniques (Bhavnani et al., 2019).

### Individualized intervention via critical games

Serious games have surfaced as potent instruments for confronting the complex challenges presented by these disorders. Developed with diagnostics, rehabilitation, and education in mind, these games offer more than mere entertainment. Operating as immersive platforms, they effectively captivate users while concentrating on particular domains of progress. In contrast to traditional approaches, serious games provide a customized and pleasurable method of intervention.

The incorporation of Piaget's theory of cognitive development into serious games enhances their complexity by enabling the development of a cognitive framework that is specific to the developmental stage of each individual. This integration represents more than just a gamification tactic; it signifies a deliberate methodology that takes into account the cognitive capacities of every participant. The progression of serious activities in accordance with Piaget's stages facilitates the development of innovative adaptive skills, thus augmenting the intervention's overall effectiveness (Elaklouk and Zin, 2019).

### Reinforcement learning: individual progress adaptation

Reinforcement learning is an additional dimension of artificial intelligence that enhances the intervention paradigm in conjunction with serious games. This type of machine learning enables an artificially intelligent agent to alter the difficulty and complexity of tasks in real time in response to the user's progress, thereby interacting dynamically with the participant's performance. A highly tailored and adaptable intervention that provides real-time responses to the specific requirements of the individual is the outcome. Reinforcement learning is a pivotal component in the domains of uncommon genetic diseases and neurodevelopmental disorders, where the range of abilities is exceptionally diverse. This guarantees that the intervention adapts to the user's progress by presenting challenges that are balanced between being overly difficult and overly easy. The ability to adapt is crucial for encouraging continued involvement and advancement in individuals who are confronted with cognitive, communication, and behavioral obstacles (Reinkensmeyer et al., 2016; Reiter et al., 2016; Manta et al., 2020; Barua et al., 2022; Nissan et al., 2023).

### Efforts to develop cognitive and communication capabilities

The amalgamation of reinforcement learning and serious games exhibits remarkable efficacy when it comes to enhancing cognitive and communicative capabilities. Conventional methods frequently employ a standardized approach, disregarding the varied requirements of individuals afflicted with rare genetic disorders. In accordance with Piaget's conceptual phases, serious games offer a sophisticated and adaptable structure for the advancement of cognition. Communication abilities, which are frequently compromised in individuals with neurodevelopmental disorders, are elevated to the center of intervention when significant games are utilized. The gamified environment fosters active engagement and participation, thereby providing an alluring setting for individuals to cultivate and refine their communication abilities. The integration of reinforcement learning guarantees that the intricacy of communication tasks increases in tandem with the user's advancement, thereby facilitating a streamlined and personalized educational journey (Malaquias and de Malaquias, 2021; Chien et al., 2022).

### Effectively handling challenging behaviors and enhancing overall quality of life

Challenging behaviors present substantial obstacles in the day-to-day functioning of people who have rare genetic diseases and neurodevelopmental disorders. Conventional behavioral interventions might prove inadequate in addressing the distinct triggers and reactions exhibited by every individual. By integrating reinforcement learning and serious games, a dynamic solution is provided that tailors behavioral interventions to the specific requirements of each individual. Through the implementation of behavioral management strategies into the form of serious games, individuals are afforded a structured and captivating setting in which to acquire knowledge and implement adaptive behaviors. The reinforcement learning element guarantees that the level of difficulty associated with challenging behaviors adapts to the user's capabilities, thereby encouraging a progressive and enduring enhancement in the ability to control such behaviors. By integrating various interventions, the overarching objective of this approach is to improve the quality of life for individuals who are afflicted with rare genetic diseases and neurodevelopmental disorders. The integration of reinforcement learning and cognitive games aims to generate significant and enduring advancements by addressing communication difficulties, cognitive deficits, and behavioral obstacles in a personalized and adaptive fashion (Verschueren et al., 2019; Beidel et al., 2021).

### An adaptive course of action

For those afflicted with neurodevelopmental disorders and rare genetic diseases, the integration of serious games and reinforcement learning ultimately represents a ray of optimism. By recognizing the distinctiveness of every person, this dynamic and adaptable strategy offers targeted interventions that surpass the limitations of conventional methods. As we contemplate the forthcoming period, this integrated paradigm presents opportunities for additional investigation, scholarly inquiry, and enhancement, thereby assuring individuals dealing with neurodevelopmental disorders and rare genetic diseases a more prospective and individualized future (Hanfstingl et al., 2019; Chetitah et al., 2023).

## Framework development: gamification and cognitive theories

This section illustrates the synergy between gamification and Piaget's theory of cognitive development, creating a framework to nurture adaptive skills in individuals dealing with rare genetic diseases and neurodevelopmental disorders. The challenges posed by these conditions call for innovative intervention strategies, and this segment delves into the amalgamation of gamification and Piaget's cognitive development theory, showcasing its role in establishing a robust structure for enhancing adaptive abilities (Toki et al., 2023).

The aim is to provide a tailored and sophisticated approach that acknowledges the diverse needs of each individual, fostering not only cognitive growth but also essential adaptive responses for an improved quality of life. Central to this integration is a comprehension of Piaget's theory of cognitive development. Piaget's theory posits that individuals progress through distinct phases marked by specific cognitive capacities and challenges, encompassing the sensorimotor, preoperational, formal operational, and concrete operational stages (Toki et al., 2023). Incorporating Piaget's theory into interventions recognizes the importance of aligning activities with an individual's current cognitive stage, facilitating more effective and targeted progress. This approach aligns with the overarching goal of cultivating adaptive skills in individuals confronting neurodevelopmental disorders and rare genetic diseases, reflecting a holistic and personalized strategy.

### Gamification: an interactive methodology for enhancing cognitive development

Gamification, when applied to serious games, offers an interactive and dynamic pathway through which Piaget's theory can be effectively implemented. Developed with educational, diagnostic, and rehabilitation purposes in mind, these games furnish players with an immersive setting in which they can engage in activities that correspond to their cognitive capacities. Instead of promoting an inert learning experience, gamification fosters active engagement, thereby enhancing the effectiveness and enjoyment of the cognitive development process. The convergence of gamification and Piaget's theory is achieved through the deliberate adaptation of game mechanics to correspond with the cognitive requirements of individual stages of development. For instance, games may emphasize fundamental motor skills and sensory exploration during the sensorimotor stage. As children advance to the preoperational stage, game-based activities may incorporate language development and symbolic representation. Games may prioritize logical reasoning and problem-solving during the concrete operational stage, whereas they may incorporate more intricate cognitive tasks during the formal operational stage (Kiesler, 2022).

### Customizing interventions via gamification

An essential benefit of incorporating gamification into Piaget's theory is the capacity to customize interventions according to the specific requirements of people afflicted with uncommon genetic disorders and neurodevelopmental diseases. Gamification, as opposed to conventional one-size-fits-all methods, facilitates personalized advancement within the game. By incorporating reinforcement learning, the level of difficulty is dynamically adjusted in real-time, thereby generating an individualized and flexible learning trajectory for every user (López-Bouzas et al., 2023).

### Establishing adaptive responses via gamification

The incorporation of gamification and Piaget's theory is not exclusively concerned with cognitive development; rather, it seeks to foster adaptive reactions in individuals confronted with these challenges. The capacity for serious games to be fully immersed enables the development of scenarios that replicate real-life obstacles. Through the process of traversing these game scenarios, players have the opportunity to cultivate and hone adaptive behaviors within a supervised and encouraging setting. Consider a scenario from a serious game intended for a preoperational infant with an uncommon genetic disorder. In keeping with Piaget's theory, the game may incorporate activities that promote symbolic representation and language development. As the child advances, the game adapts dynamically by incorporating increasingly difficult tasks that simultaneously test and improve cognitive and adaptive abilities. An additional illustration could pertain to an adolescent during the formal operational phase, during which the game integrates sophisticated problem-solving exercises that replicate real-life obstacles. By adjusting the level of difficulty of these tasks in accordance with the adolescent's performance, the reinforcement learning component generates an optimal and adaptable learning environment. In addition to its influence on cognitive development, the incorporation of gamification and Piaget's theory significantly affects the quality of life for those afflicted with neurodevelopmental disorders and uncommon genetic diseases. Enhancing adaptive abilities through the engagement and enjoyment of games enables individuals to more effectively confront the obstacles they encounter on a daily basis. Conversely, this promotes a more nurturing and optimistic atmosphere by mitigating the strain on family members and caregivers (Means and Neisler, 2023).

### Challenges and factors to be considered

Although the amalgamation of gamification and Piaget's theory presents a potentially fruitful strategy, it is not devoid of obstacles. The process of modifying games to suit the varied requirements of individuals at different phases of development necessitates meticulous planning and ongoing improvement. The preservation of accessibility and inclusivity for people with diverse abilities is of the utmost importance. Further investigation is warranted with regard to ethical considerations pertaining to data privacy and the potential ramifications of prolonged screen usage. Building upon this theoretical exploration, the amalgamation of gamification with Piaget's theory emerges as a promising strategy. However, this approach is not without its challenges and considerations. The process of modifying games to accommodate the diverse requirements of individuals at different developmental phases necessitates meticulous planning and continuous improvement. Ensuring the preservation of accessibility and inclusivity for people with diverse abilities becomes paramount in this context. Moreover, as we plunge into the practical implementation, further investigation is warranted, particularly concerning ethical considerations related to data privacy and the potential consequences of prolonged screen usage (Aloizou et al., 2021).

### Prospects for future directions and potential research pathways

As the integration of gamification and Piaget's theory is further explored, a multitude of prospects for future research come to light. It is crucial to investigate the prospective effects of these interventions on the development of cognitive and adaptive abilities. Conducting research on the efficacy of these interventions in a wide range of neurodevelopmental disorders and rare genetic diseases will aid in the development and expansion of this strategy. Furthermore, gaining insight into the inclinations and encounters of players involved in serious games will contribute to the development of interventions that are both more efficient and intuitive for users. In summary, the amalgamation of gamification and Piaget's theory of cognitive development presents a robust framework for fostering adaptive abilities in patients afflicted with neurodevelopmental disorders and uncommon genetic diseases. The integration of theory and technology in this symbiotic manner facilitates an individualized, captivating, and flexible method of intervention (McLeod, 2018). By recognizing and embracing the wide range of cognitive abilities that exist during various phases of development, we enable ourselves to access the possibility of significant advancement and adjustment. In the pursuit of understanding the intricacies of neurodevelopmental disorders and rare genetic diseases, this integrated approach presents a dynamic trajectory that promotes adaptive responses that are vital for enhancing the overall quality of life and not only cognitive development.

## Critical discussion: implications and future research

While our exploration of the integration of reinforcement learning and serious games has shed light on their transformative potential for individuals facing rare genetic diseases and neurodevelopmental disorders, it is essential to draw parallels with the study's outcomes on cognitive development through play. The study infers that, akin to our discussion on technology-assisted interventions, age does not significantly impact the cognitive benefits derived from play; rather, it is the duration of engagement that plays a pivotal role. This aligns with the broader understanding that technology, like play, can serve as a catalyst for cognitive enhancement, offering valuable insights into tailored and dynamic interventions (Bormanaki and Hoshhal, 2017).

As we delve into the transformative potential of the integrated methodology in our discourse, the study's findings resonate with the idea that dedicating more time to play correlates with heightened cognitive abilities. Similarly, the integration of reinforcement learning and serious games strives to enhance adaptive responding and cognitive development, emphasizing the role of continuous improvement and adaptation. Both discussions converge on the importance of ongoing development, whether in the technological or play context, to unlock the full potential for individuals facing complex challenges. This parallel underscores the imperative for further investigation, development, and expansion in both realms, acknowledging the evolving nature of these interventions and their significant impact on individuals' wellbeing.

### Consequences resulting from integration

The incorporation of reinforcement learning and serious games into intervention strategies for rare genetic diseases and neurodevelopmental disorders has significant ramifications. By merging the educational and thought-provoking aspects of serious games with the flexibility of reinforcement learning, a personalized and dynamic intervention model is formulated. A noteworthy implication lies in its ability to overcome the constraints associated with conventional, one-size-fits-all methodologies. This integrated approach, acknowledging and catering to the distinct needs and developmental stages of each individual, establishes a more tailored and effective support system.

These implications extend beyond cognitive growth, encompassing comprehensive personal development, adaptive responses, and behavior regulation. Aligned with Piaget's theory, serious games offer a structured environment conducive to cognitive development. Concurrently, reinforcement learning introduces adaptive challenges that foster the acquisition of essential life skills. Therefore, by embracing this integrated approach, it becomes possible not only to address the specific challenges associated with these disorders but also to nurture a holistic set of abilities essential for everyday life. Additionally, the integration of reinforcement learning and serious games offers a promising avenue for supporting caregivers. The personalized and dynamic nature of this approach means that caregivers can benefit from targeted interventions that cater to the unique needs and developmental stages of the individuals they are supporting. This not only enhances the effectiveness of caregiving but also provides caregivers with valuable tools and strategies to navigate the complexities associated with rare genetic diseases and neurodevelopmental disorders. As caregivers play a pivotal role in the support network, the integration of these technologies can contribute to a more comprehensive and empowering caregivers' experience (Stasolla et al., 2019).

### Clinical applications

There are numerous potential clinical applications for the integration of reinforcement learning and serious games. Initially, this integrated methodology serves as a tool for a comprehensive evaluation, encompassing cognitive capacities, communicative proficiencies, and behavioral control. While studies on cognitive development primarily focus on addressing issues related to the emergence of new cognitive structures, there is a secondary aspect that tends to be overlooked (McLeod, 2018). Similar to the biological process of dissimilation and the dissipation observed in open systems in physics, achieving adequate cognitive adaptation to the environment requires not only the development of new schemas but also the exclusion of non-adaptive components. This paper aims to shed light on this neglected aspect and discusses two potential methods for excluding the “wrong” components. Since these components are either useless or act as hindrances to the organism's responsiveness to environmental demands, their exclusion can be characterized either as extinction or as an active process of dissimilation. The paper specifically searches into cognitive dissimilation concerning the challenge of blocking the activation of impedimental components. Additionally, it underscores the relationships between cognitive dissimilation and the processes of assimilation and accommodation as conceptualized in Piaget's theory of cognitive development, pointing out the need for further exploration in this area (Dodonov and Dodonova, 2011; Zhang et al., 2021).

Reinforcement learning facilitates dynamic adaptation, empowering clinicians to collect real-time data on individual progress. This enables a more comprehensive understanding of the participant's strengths and areas that may require additional assistance. This integrated approach proves to be a valuable resource in diagnosing and monitoring neurodevelopmental disorders, furnishing clinicians with a comprehensive perspective of the individual's abilities and obstacles. Moreover, the level of involvement fostered by serious activities during therapeutic interventions significantly increases. This engagement is critical in a clinical setting to ensure consistent participation, a critical determinant of intervention efficacy. Reinforcement learning facilitates real-time adaptation, guaranteeing that therapeutic activities remain both demanding and attainable, accommodating the changing requirements of the individual. This not only enhances the participant's experience but also enables a more precise assessment of their development and aptitudes (Dodonov and Dodonova, 2011). The incorporation of reinforcement learning techniques into clinical environments additionally provides opportunities for the ongoing refinement of therapeutic approaches. The ability of the artificial intelligent agent to modify tasks in response to individual progress enables clinicians to scrutinize and assess the efficacy of various methodologies. The application of this adaptive learning cycle facilitates the continuous improvement of interventions, aiding in the formulation of therapeutic strategies that are both more efficacious and individualized (Stasolla et al., 2022).

## Assessment and training of executive functions in children through a game-based software

Further elaborate on the introductory account, the manuscript and Nappo et al. (2022) offers a thorough examination of the novel application of game-based software in evaluating and instructing children's executive functions (EFs), with a particular focus on individuals afflicted with neurodevelopmental disorders. Attention, inhibition, working memory, cognitive flexibility, and planning are examples of EFs that are essential for adaptive and goal-directed behavior. In their study, Nappo et al. (2022) examine the intricacies of the ASTRAS software, which was developed and designed with the explicit purpose of evaluating and instructing EFs in children. This study highlights the criticality of integrating feedback from both clinicians and children in order to optimize the software's usability and clinical validity by accommodating their preferences and requirements. One notable feature of ASTRAS is its implementation of gamification, wherein conventional cognitive exercises are converted into captivating and pleasurable games, thus fostering greater motivation and engagement among children in therapeutic endeavors.

The software's design demonstrates a heightened awareness of the clinicians' requirement for all-encompassing assessment tools in order to precisely characterize the cognitive functioning of a patient and devise efficacious rehabilitation programs. By conducting an assessment session, ASTRAS enables this by generating a comprehensive profile of a child's executive functioning and identifying the specific domains that necessitate intervention. Furthermore, ASTRAS incorporates the expanding practice of telerehabilitation, which enables therapists to delegate assignments to children for completion at home, thus expanding the scope of therapy beyond conventional clinical environments. According to the research conducted by Nappo et al. (2022), therapists regarded ASTRAS as a user-friendly and clinically suitable tool for evaluating and instructing their patients' EFs. The positive reception of the software among children, who exhibited a predilection for training activities rather than assessment tasks, demonstrates the efficacy of gamification in sustaining their interest. The favorable response from both therapists and children highlights the potential of ASTRAS as a beneficial instrument in the domain of cognitive development and rehabilitation. It presents an innovative and captivating strategy for enhancing executive functions in children who are afflicted with neurodevelopmental disorders.

Chu et al. (2021) explored the effects of a concept-effect relationship and an interactive game-based learning system as an useful tool for the organization learning material in developing a diagnostic and remedial system for detecting students' learning problems. An experiment was conducted an elementary school mathematics course to evaluate the outcomes of proposed approach. Data demonstrated an improvement of learning achievement. Furthermore, it enhanced learning attitudes and self-efficacy, reducing their cognitive load in mathematics course.

### Prospects for future research and development

The ongoing integration of reinforcement learning and serious games in the context of rare genetic diseases and neurodevelopmental disorders presents substantial opportunities for research and development. Firstly, it is crucial to systematically assess the enduring effects of this integrated methodology on cognitive, adaptive, and behavioral development. Understanding both the lasting advantages and potential obstacles over extended periods is essential to enhance and validate its efficacy. Furthermore, comprehensive research is necessary to explore the broad implications of this integrated methodology for various neurodevelopmental disorders and rare genetic diseases. Ensuring adaptability and efficacy requires tailoring interventions to the unique attributes of specific conditions. Comparative studies across disorders will enhance comprehension of the approach's potential uses and constraints. As sophisticated technologies become integral to clinical interventions, ethical considerations demand meticulous examination. Privacy concerns, consent procedures, and the potential ramifications of prolonged screen usage require thorough exploration. Further investigation is warranted to develop ethical principles and optimal methodologies for the responsible integration of these technologies in clinical environments.

Continuous development efforts should prioritize enhancing the algorithms and learning models used in reinforcement learning, aiming for real-time adaptability to customize interventions further. Collaboration among researchers, clinicians, and technology developers is essential to propel the field forward. In summary, the amalgamation of reinforcement learning and serious games has the potential to revolutionize interventions for those with neurodevelopmental disorders and rare genetic diseases. Rigorous examination and practical implementations demonstrate the capability of this approach to deliver sophisticated and efficient assistance. However, ongoing development must confront obstacles, perfect ethical deliberations, and broaden its scope to encompass a variety of disorders. The collaborative efforts of technology developers, clinicians, and researchers are vital for a paradigm shift, providing adaptable, individualized, and empowering interventions.

## Conclusion

Within the complex domain of rare genetic diseases and neurodevelopmental disorders, an innovative approach that combines reinforcement learning with serious games presents itself as a viable solution. This integration has the potential to provide individuals with these challenges with transformative support. By integrating serious games with Piaget's theory of cognitive development, a customized intervention is created that caters to the varied requirements of individuals at different stages of cognitive development. By adopting this comprehensive and personalized approach, one not only promotes cognitive development but also nurtures adaptive responses, thereby strengthening the overall framework. The integrated methodology facilitates customized learning experiences within serious games by employing reinforcement learning to dynamically adapt to the unique progress of each participant. The personalized approach described here goes beyond the standard one-size-fits-all approach, fostering an environment that is conducive to the growth and development of adaptive skills that are essential for everyday existence. Moreover, this individualized support extends beyond cognitive spheres, influencing how individuals navigate daily challenges and contributing to a more fulfilling and empowered life.

The integrated approach described here has a profound and far-reaching effect on the quality of life for individuals as a whole, thereby mitigating the strains placed on families and caregivers. Recognizing the obstacles encountered by individuals providing care, the integrated approach endeavors to mitigate their workload by implementing dynamic and efficacious interventions that foster caregiver empowerment and cultivate a sense of optimism. This not only transforms the lives of individuals facing rare genetic diseases but also elevates the wellbeing of those who selflessly provide support. Redefining the intervention landscape, the integration of reinforcement learning and serious games not only provides assistance but also a transformative experience for individuals and their support networks but also serves as a dynamic path forward. This innovative methodology serves as an inspiration, fostering development and adjustment, offering a look into a future characterized by increased empathy and agency for families, caregivers, and individuals. As this integrated approach continues to evolve, it holds the promise of reshaping the narrative around rare genetic diseases and neurodevelopmental disorders, steering toward a future where individuals thrive, caregivers find empowerment, and families experience a newfound sense of hope and resilience (Stasolla et al., 2023a,b).

## Author contributions

FS: Writing – review & editing, Conceptualization. KA: Writing – original draft. AP: Writing – review & editing, Validation, Supervision. MD: Writing – review & editing, Validation, Supervision. MDG: Writing – review & editing, Supervision. AZ: Writing – review & editing.
